# Identification of single-stranded and double-stranded dna binding proteins based on protein structure

**DOI:** 10.1186/1471-2105-15-S12-S4

**Published:** 2014-11-06

**Authors:** Wei Wang, Juan Liu, Xionghui Zhou

**Affiliations:** 1 School of Computer, Wuhan University, Wuhan, Hubei 430072, People's Republic of China

## Abstract

**Background:**

Protein-DNA interactions are essential for many biological processes. However, the structural mechanisms underlying these interactions are not fully understood. DNA binding proteins can be classified into double-stranded DNA binding proteins (DSBs) and single-stranded DNA binding proteins (SSBs), and they take part in different biological functions. DSBs usually act as transcriptional factors to regulate the genes' expressions, while SSBs usually play roles in DNA replication, recombination, and repair, etc. Understanding the binding specificity of a DNA binding protein is helpful for the research of protein functions.

**Results:**

In this paper, we investigated the differences between DSBs and SSBs on surface tunnels as well as the OB-fold domain information. We detected the largest clefts on the protein surfaces, to obtain several features to be used for distinguishing the potential interfaces between SSBs and DSBs, and compared its structure with each of the six OB-fold protein templates, and use the maximal alignment score TM-score as the OB-fold feature of the protein, based on which, we constructed the support vector machine (SVM) classification model to automatically distinguish these two kinds of proteins, with prediction accuracy of 87%,83% and 83% for HOLO-set, APO-set and Mixed-set respectively.

**Conclusions:**

We found that they have different ranges of tunnel lengths and tunnel curvatures; moreover, the alignment results with OB-fold templates have also found to be the discriminative feature of SSBs and DSBs. Experimental results on 10-fold cross validation indicate that the new feature set are effective to describe DNA binding proteins. The evaluation results on both bound (DNA-bound) and non-bound (DNA-free) proteins have shown the satisfactory performance of our method.

## Background

The family of DNA binding proteins is able to recognize and bind to DNAs, and they play vital roles in many biological processes such as DNA replication, recombination, repair, transcription, translation, and maintenance of telomeres, and so on [1-4]. There are two kinds of DNAs, single-stranded DNA (ssDNA) and double-stranded DNA (dsDNA). Accordingly, the DNA binding proteins usually consist of single-stranded DNA-binding proteins (SSBs) and double-stranded DNA-binding proteins (DSBs). SSB binds with ssDNA with high affinity and low specificity, and is mainly involved in DNA replication, recombination and repair. While DSBs involve in binding to particular dsDNA sequences, to modulate the process of transcription, to cleave DNA molecules, or to be involved in chromosome packaging and transcription in the cell nucleus, etc. Though there are some researches [5-7] on the SSB and DSB respectively, few attentions have been paid on investigating what makes SSB and DSB have such different kind of binding specificity.

With the development of biotechnology, a large amount of proteins have been sequenced. However, SSBs have shown to have little sequence conservation [8]. Even DSBs involved in similar functions may have conserved subsequences, different kinds of DSBs with different functions seems to show few common subsequences. Therefore, it is hard to recognize SSB sequences from DSB sequences, or vice versa. Now that the molecular structure determines its biological function, structural information is expected to provide insight on the binding mechanism of SSB or DSB. The great progress of the structure genomics project [9] results that more and more high resolution 3D structures for DSBs and SSBs are available now, which makes it possible to investigate the common structural differences between SSB and DSB that are responsible for the binding specificity. In the meantime, the investigation results can help to annotate or refine the annotation of the proteins with known structures yet unknown or not fully understood functions. In fact, up to Jan. 25, 2013, the Protein Data Bank (PDB) [10] contains 3390 structures for DNA binding proteins (see Additional file 1), among them only about 30% and 5% are annotated as DSBs and SSBs, respectively, and whether the remains belong to DSBs or SSBs are still not very clear. Therefore, a computational method is required to annotate the DNA binding protein as DSB or SSB automatically. To address this question, this work is devoted to characterize the structural differences between DSBs and SSBs, and then to construct the distinguishing model that can automatically refine the annotations of the DNA binding proteins.

The surface of a protein is generally irregular, containing many clefts and grooves of varying shapes and sizes [11]. Previous researches have shown that a large cleft can provide an increased opportunity for the protein to form interactions with other molecules, particularly small ligands [12,13]. Therefore, some researches used a particularly large and deep cleft to characterize the binding active sites of the proteins [11,13,14]. We guess that for DNA binding proteins, the cleft properties on the surface may also play important roles on the dsDNA/ssDNA binding specificity.

Research results have shown that although the sequences of different SSBs are very different, there are well-conserved elements in the structures. That is, most SSBs contain one or more OB (oligonucleotide/oligosaccharide binding) -fold domains [6,15-18]. A typical OB-fold has a five-stranded beta-sheet coiled to form a closed beta-barrel. This barrel is capped by an alpha-helix located between the third and fourth strands. The OB-fold plays critical role in binding with ssDNA. Although it is hard to say that the OB-fold is unique for SSBs, we think that it should also be used as an important descriptor to distinguish SSBs from DSBs.

In this paper, we aim to investigate the structural differences between collected SSBs and DSBs, and extract the structure-based features related to surface clefts and OB-folds, based on which, we construct a computational model that can automatically classify the DNA protein as a DSB or SSB by using the widely used support vector machine (SVM). The promising performance suggests that our method will be useful in the protein function annotation and refinement.

## Methods

### Data sets

We first extracted the structures of all 3390 DNA binding proteins from PDB (Jan. 25, 2013 release) according to their annotations, which contain 1039 DSBs (HOLO 890, APO 149), 158 SSBs (HOLO 70, APO 88) and 2193 unknowns. Then we use PISCES (http://dunbrack.fccc.edu/PISCES.php) [19] to get the non-redundant set, in which every structure is either solved by NMR or by X-ray yet with resolution better than 3Å, the sequence identity is less than 30%, and the length of chain is greater than 40 amino acid residues. As a result, we finally got 204 DSBs (HOLO 154 and APO 50), 75 SSBs (HOLO 37 and APO 38) and 727 unknowns (Additional file 2). For simplicity, we call the set containing protein-DNA bound structures as HOLO set, and the set containing protein-DNA unbound structures as APO set, and the proteins in these sets are respectively denoted as DSB_holo, SSB_holo, DSB_apo, and SSB_apo hereinafter.

### Features on clefts

The protein surface has a very complex and irregular shape that contains concave, convex and flat, which contributes to protein to interact with the external environment. The clefts, pockets, or cavities are generally considered as the active sites on protein surfaces, thus the research on them are meaningful of understanding the protein functions.

Now that it has been reported that a large cleft can provide an increased opportunity for the protein to form interactions with other molecules [12,13], and the particularly large and deep clefts have been used to characterize the binding sites of the proteins [11], we consider that for DNA binding proteins, the large clefts on the surface may also play important roles on the dsDNA/ssDNA binding. In other words, the large clefts on SSB would be narrow enough to prevent it from binding with dsDNA.

Some tools have been developed to recognize the clefts based on the protein structures, such as HOLE [20], MOLE [21,22], MolAxis [23] and Caver [24,25]. In this work, we applied CAVER 3.0 package to detect the clefts and the corresponding indexes of the largest clefts (also called as tunnels in this work) on the protein surfaces, to investigate whether they are possible to be used for distinguishing the potential interfaces between SSBs and DSBs. Concretely, we mainly got three indexes of the detected tunnels: length, curvature and bottleneck radius.

**Length: **indicating the length of the path from the start point to the end point along the tunnel axis.

**Curvature: **indicating the curvature of the tunnel. The curvature of the tunnel is calculated by Curvature = Length/Distance, where the distance is the length of the straight line from the start point to the end point of the tunnel. The greater the curvature, the curved is the tunnel.

**Bottleneck radius: **indicating the radius of the narrowest part of the tunnel, also representing the radius of the largest possible ball that can be centered at a given point of the tunnel axis without colliding with the input structure.

**Figure 1:**
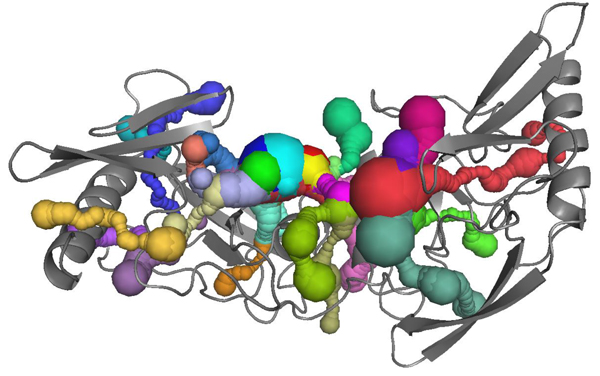
**All detected tunnels of protein 1A73**. The graph shows the CAVER package detects out 27 tunnels in 1A73 protein, and show 3D structure for all tunnels with different colours in protein surface.

**Figure 2:**
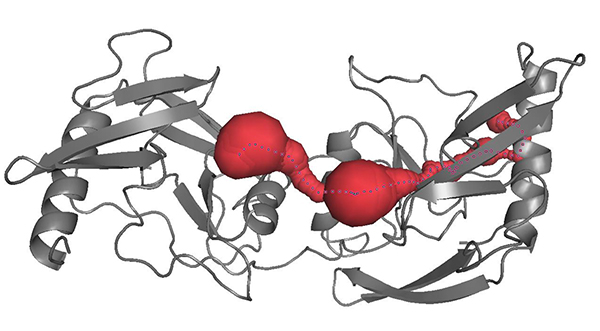
**The largest tunnel (25#) of protein 1A73**. The graph shows the red tunnel is the largest tunnel in terms of maximizing (Length*Bottleneck Radius).

Since the protein surface contains many tunnels of varying shapes and sizes. The CAVER package return as many tunnels as possible. For the reason mentioned above, we just check the largest one in terms of maximizing (Length*Bottleneck Radius). For example, for protein 1A73, CAVER detects out 27 tunnels shown in Figure 1, and 1their indexes are listed in Table 1. According to the choosing criteria, tunnel number 25 (Figure 2) will be considered as the largest tunnel.

**Table 1:** Index values for all tunnels of 1A73

Tunnel	Bottleneck-radius	Length	Curvature
1	3.52	2.47	1.05
2	2.79	3.48	1.26
3	2.54	7.64	1.26
4	1.85	5.85	1.77
5	1.86	12.08	2.02
6	1.33	14.78	1.29
7	1.25	12.68	1.43
8	0.96	13.08	1.39
9	1.09	15.63	1.50
10	1.13	16.26	1.71
11	1.03	29.47	1.57
12	0.98	25.02	1.62
13	1.03	35.71	1.61
14	1.07	33.06	2.00
15	0.77	19.99	1.43
16	0.77	35.07	1.47
17	0.79	25.53	2.09
18	0.71	24.74	1.39
19	0.77	28.35	1.32
20	0.72	38.97	1.78
21	0.88	51.54	1.62
22	0.70	46.82	1.47
23	0.77	36.59	1.40
24	0.73	41.06	1.47
**25**	**0.74**	**62.01**	**1.64**
26	0.72	45.18	3.11
27	0.72	47.09	2.18

This table shows the values of bottleneck radius, length and curvature for the all tunnels. Note that the boldface (25#) presents the values of the largest tunnel.

### Feature on OB-fold domain

OB-fold is a small structural motif that was first characterized in 1992 in four proteins that bind either oligonucleotides or oligosaccharides [26]. Typically, the OB fold comprises a five-stranded β-sheet coiled to form a closed β barrel and capped by an α-helix located between the third and fourth β strands [27-30]. Although OB-fold has since been observed at protein/protein interfaces as well, but the nucleic acid-binding superfamily is the largest within the OB-folds, and proteins containing OB-folds involve almost any time that single-stranded DNAs or RNAs are present or require manipulation [8]. Now that OB-folds are conserved and play important roles in SSB-ssDNA binding, we extract the feature indicating whether OB-fold is contained in a protein, with the hope that the feature is able to distinguish SSBs with DSBs.

**Figure 3:**
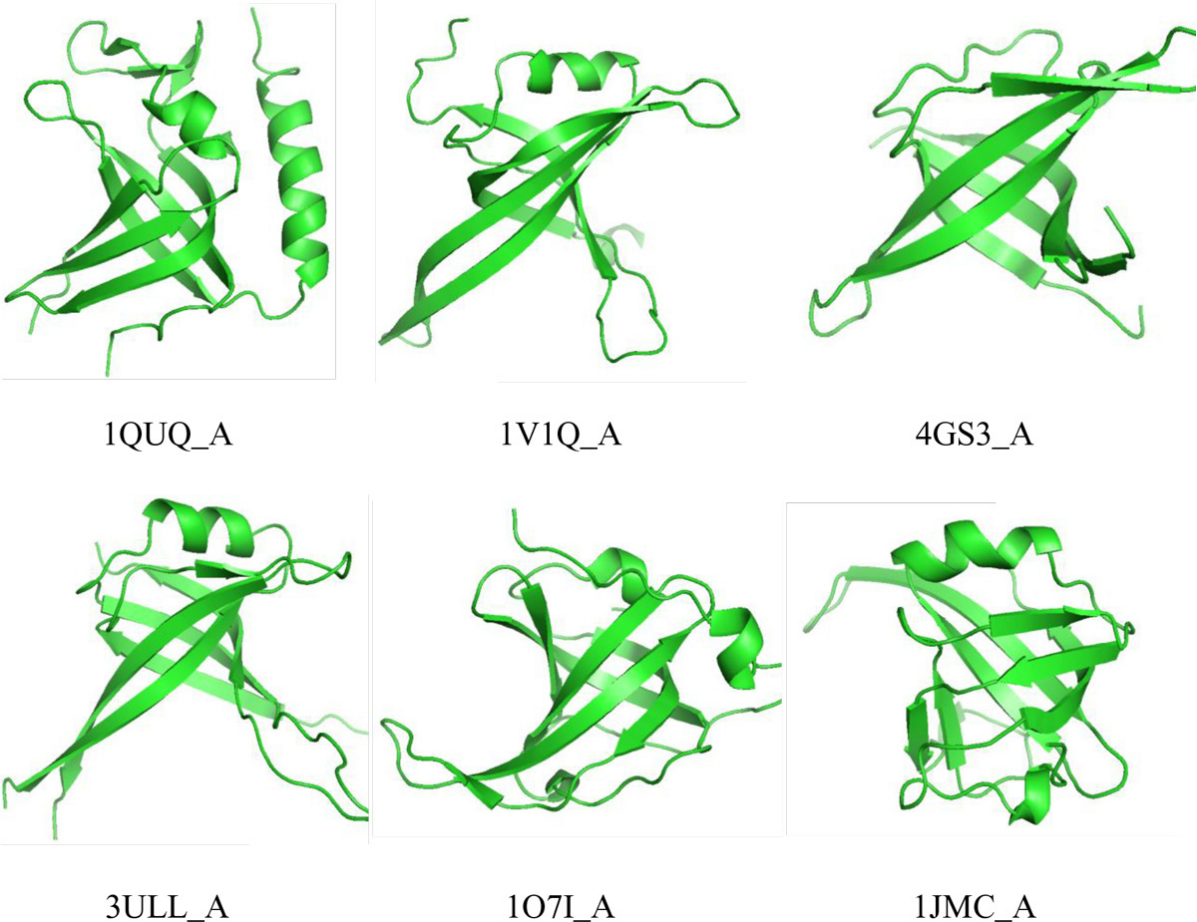
**Six templates of the OB-fold domain**. They show structural similarity but different topologies, and the similarity of sequences are with <30%.

Considering that OB-folds evolve into several variants though they are very conserved, we choose the chain A of six typical proteins (PDB:1QUQ [31], 1V1Q [32], 4GS3 [33], 3ULL [34], 1O7I [35], 1JMC [36]) shown in Figure 3 as OB-fold templates. From Figure 3, we can see that these proteins contain nothing except for OB-fold domains. Moreover, each chain of the former five proteins contains one and only one OB-fold domain. Since 1JMC_A contains two OB-fold domains, we only use one of them as the template.

For an unknown protein, we use the protein structure alignment package TM-align [37] to compare its structure with each of the templates and use the maximal alignment score TM-score as the OB-fold feature of the protein.

### Classification model and evaluation

In this work, we used support vector machine (SVM) to build the classification model. The SVM classifiers were implemented using Matlab 2012a SVM package with the Gaussian Radial Basis Function (RBF) as a kernel.

In order to evaluate the performance of the prediction results, we used several measures, including Accuracy, Sensitivity, Specificity, and F-measured and area under the receiver operating characteristic curve (AUC). Let TP (true positive) is the number of proteins correctly predicted as SSBs, FP (false positive) is the number of proteins incorrectly predicted as SSBs, TN (true negative) be the number of proteins correctly predicted as DSBs and FN (false negative) be the number of proteins incorrectly predicted as DSBs. The accuracy (ACC), sensitivity (SN), specificity (SP), F-measured (F1) and Matthews Correlation Coefficient (MCC) are defined as the following:

(1) $$\text{Accuracy}=\;\frac{\left(TP\;+\;TN\right)}{\left(TP\;+\;FN\;+\;TN\;+\;FP\right)}$$

(2) $$\text{Sensitivity}=\frac{TP}{\left(TP\;+\;FN\right)}$$

(3) $$\text{Specificity}=\frac{TN}{\left(TN\;+\;FP\right)}$$

(4) $$\text{F - measure}=\frac{2\times TP}{2\times TP+FP+FN}$$

(5) $$\text{MCC}=\frac{TP\times TN-FP\times FN}{\sqrt{\left(TP+FP\right)\times \left(TP+FN\right)\times \left(TN+FP\right)\times \left(TN+FN\right)}}$$

We use 10-fold cross validation test to evaluate the classification performance. Because of the unbalance of different kinds of proteins, in each fold we iterate 15 times to randomly select the equal numbers of SSBs and DSBs into the train set by using down-sampling method, and use the voting strategy to assign the class label of the test protein. To the best of our knowledge, there is no computational method to distinguish SSBs from DSBs, therefore we also train the random classifier as the baseline in each test.

## Results and discussion

### Investigation of the distinguishing ability of the features

**Figure 4:**
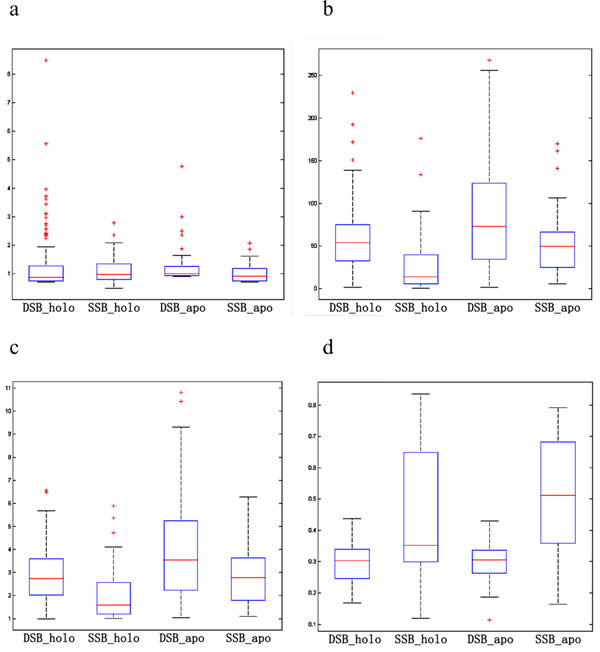
**Feature distributions of different kinds of DNA-binding proteins**. These graphs show the box plot of the four features for the HOLO and APO datasets. Those are (a) tunnel bottleneck radius, (b) tunnel length, (c) tunnel curvature and (d) TM-score.

By using CAVER3.0, we have detected 990 tunnels from HOLO set (865 for DSBs, 125 for SSBs), and 1168 tunnels from APO set (757 for DSBs, 411 for SSBs). According to the maximizing criterion described above, we selected one maximal tunnel for each protein. As a result, we finally got 37 tunnels for bound (DNA-bound) SSBs, 38 tunnels for unbound (DNA-free) SSBs, 154 tunnels for bound DSBs and 51 tunnels for unbound DSBs. Accordingly, we also got three feature values for each tunnel. By using TM-align, we aligned every protein with each of the six OB-fold templates shown in Figure 3, and got the maximal alignment score as the TM-score of the protein. In order to investigate the distinguishing ability of the features, we had statistically analysed the distribution for each feature, shown in Figure 4. It is obvious that, bottleneck radius shows little difference between DSBs and SSBs in either bound or unbound forms; and the DNA binding protein in bound form tends to have larger bottleneck radius than that in unbound form, which may be due to the fact that the protein usually need to widen the tunnel for binding with the DNA. SSBs tend to have the smaller tunnel length and curvature than DSBs, and tunnel length seems to be more distinguishable than tunnel curvature between DSBs and SSBs; moreover, it seems easier to differentiate DSBs and SSBs in bound forms than in unbound forms by using either of the features. As expected, SSBs obtain much higher TM-scores than DSBs by comparing to the OB-fold templates, illustrating that most SSBs have OB-fold like domains. In conclusion, TM-score, tunnel length and tunnel curvature are usable features to construct distinguish model for SSBs and DSBs, while bottleneck radius is lack of the distinguishing ability. Since the statistical results of tunnel length and tunnel curvature are very similar, we further investigate the correlation between these two features, listed in Table 2 showing that they are actually positive correlated with each other.

**Table 2:** Correlation of tunnel length and tunnel curvature

Dataset	Protein types	Pearson-coefficient	P-value
HOLO set	DSBs	0.6929	2.3752e-23
	SSBs	0.4484	0.0054
APO set	DSBs	0.9293	7.7890e-23
	SSBs	0.5599	2.5705e-04

This table shows the values of Pearson coefficient and P-value between tunnel length and curvature. The columns of Pearson coefficient and P-value correspond to the pairs of DSBs/SSBs in HOLO set and APO set, respectively.

### Validation of the differentiating features

We have done the validation experiments on HOLO set and APO set by using one, two or three features to construct the classification models. The validation performances are shown in Table 3, 4 respectively. From the tables we can see that, feature TM-Score can recognize out SSBs with high accuracy, while the feature tunnel length/curvature can recognize out DSBs with high accuracy, meaning that the distinguishing abilities of TM-Score and length/curvature are complementary. The performance of the classification model constructed with length feature is better than that constructed with curvature, also better than or nearly equal to that constructed with length and curvature features, further confirming that curvature feature is redundant with length feature and adding redundant features into the classification model does not necessarily get the positive response. Compared to the model with single feature, the significant enhancement of performance when using TM-Score together with one or more other features showing that constructing classification models with complementary features is preferable to the discrimination of DSBs and SSBs.

**Table 3:** Performance on HOLO set

Feature		ACC	SN	SP	AUC	MCC	F1
Length		0.7470	0.7725	0.7258	0.7539	0.5207	0.7681
Curvature		0.6808	0.7058	0.6525	0.6818	0.3760	0.6949
TM-Score		0.7054	1.0000	0.4050	0.6629	0.5018	0.7823
Length+Curvature		0.7725	0.7925	0.7508	0.7738	0.5633	0.7889
Length+TM-Score		0.8434	0.8308	0.8583	0.8476	0.7012	0.8472
Curvature+TM-Score		0.7824	0.7750	0.7925	0.7866	0.5848	0.7903
Length+Curvature+TM-Score		0.8686	0.8725	0.8608	0.8710	0.7497	0.8782
Baseline	Random 1 feature	0.5040	0.5082	0.4859	0.4967	-0.0056	0.5553
	Random 2 features	0.4951	0.4907	0.5137	0.5028	0.0040	0.5697
	Total 3 features	0.4938	0.4914	0.5036	0.4968	-0.0043	0.5789

**Table 4:** Performance on APO set

Feature		ACC	SN	SP	AUC	MCC	F1
Length		0.6533	0.4783	0.8267	0.6548	0.3205	0.5732
Curvature		0.5487	0.3033	0.8000	0.5676	0.1119	0.4005
TM-Score		0.8015	0.9525	0.6483	0.8117	0.6424	0.8425
Length+Curvature		0.6401	0.4658	0.8192	0.6491	0.3101	0.5689
Length+TM-Score		0.8543	0.9800	0.7242	0.8511	0.7378	0.8848
Curvature+TM-Score		0.8518	0.9850	0.7167	0.8592	0.7365	0.8836
Length+Curvature+TM-Score		0.8310	0.9533	0.7058	0.8286	0.6934	0.8620
Baseline	Random 1 feature	0.4990	0.4991	0.4988	0.4990	-0.0025	0.4712
	Random 2 features	0.4991	0.4973	0.5016	0.4998	-0.0014	0.4875
	Total 3 features	0.5019	0.5054	0.4973	0.5019	0.0028	0.5035

### Independent test on APO set

In many cases, it is easier to collect information on DNA binding proteins in the bound form than in unbound form, whereas we need to know whether an unknown unbound protein be SSB or DSB. Thus, we train the classifier on HOLO set and test it on APO set. The results are listed in Table 5 from which we can see that the structural information on tunnel and OB-fold can actually reflect that differences between SSBs and DSBs thus can be used as discriminant features to build the classification model.

**Table 5:** Performance of the independent test

Feature		ACC	SN	SP	AUC	MCC	F1
Length+TM-Score		0.7191	0.8235	0.5789	0.7012	0.7097	0.4179
Curvature+TM-Score		0.6854	0.7451	0.6053	0.6752	0.6389	0.3531
Length+Curvature+TM-Score		0.7303	0.7647	0.6842	0.7245	0.6842	0.4489
Baseline	Random 1 feature	0.5009	0.5059	0.4943	0.4997	0.0004	0.4927
	Random 2 features	0.5011	0.5014	0.5007	0.5010	0.0022	0.5312
	Total 3 features	0.5006	0.5041	0.4958	0.5007	-0.0002	0.5323

### Prediction on mixed set

In practice, we often found the available dataset include not only the bound form proteins, but also the unbound form proteins, whereas we need to know whether an unknown DNA binding protein be SSB or DSB. Thus, we have done the validation experiments on the mixed set by using one, two or three features construct the classification models. The results are listed in Table 6 from the tables we can see that, feature TM-Score can still recognize out SSBs with high accuracy in each single feature. Compared to the models with single feature, the best performance using more features with an accuracy of 0.8251, MCC of 0.6632, SN of 0.8605 and SP of 0.7904 is much better. Thus, we further train the classifier on mixed set and predicted the unknown proteins (727 unknowns). The classified results are listed in additional file 2.

**Table 6:** Performance on mixed set

Feature		ACC	SN	SP	AUC	MCC	F1
Length		0.6161	0.7132	0.5170	0.6237	0.2446	0.6548
Curvature		0.5984	0.6887	0.5063	0.6097	0.2018	0.6374
TM-Score		0.7558	0.9846	0.5230	0.7571	0.5756	0.8104
Length+Curvature		0.6114	0.7004	0.5211	0.6169	0.2300	0.6457
Length+TM-Score		0.7984	0.8880	0.7089	0.8076	0.6222	0.8259
Curvature+TM-Score		0.7701	0.9407	0.5980	0.7802	0.5844	0.8133
Length+Curvature+TM-Score		0.8251	0.8605	0.7904	0.8265	0.6632	0.8372
Baseline	Random 1 feature	0.5030	0.5070	0.4918	0.4992	-0.0011	0.5201
	Random 2 features	0.4996	0.4986	0.5024	0.5001	0.0013	0.5369
	Total 3 features	0.5014	0.5021	0.4992	0.5007	0.0015	0.5525

## Conclusion

Despite many similar properties, dsDNA and ssDNA possess distinctive entities that are recognized differently by specialized dsDNA and ssDNA binding proteins, respectively. SSBs and DSBs binding interfaces are thus expected to differ in their geometrical features consistent with the different nature of dsDNA and ssDNA [29,38,39]. While the sequence and structural properties of DSBs and SSBs binding interfaces has been studied during the last decade [28,40], computationally distinguishing between the DSBs and SSBs binding interfaces is still a lack of research. In this study, we investigated surface tunnels features of SSBs and DSBs and found that they have different ranges of tunnel lengths and tunnel curvatures; moreover, the alignment results with OB-fold templates have also found to be the discriminative feature of SSBs and DSBs. Therefore, we made the first try to present a method to computationally distinguish SSBs with DSBs based on the discriminant features and got the satisfactory results.

The protein surface features should also be useful for the analysis of other types of molecular interactions, such as protein-ligand, protein-RNA, and protein-protein complexes, and for the study of a variety of proteins, multiple binding sites or a specific family of proteins. These problems would require modelling interface surfaces of different characteristics such as compatibility, different sizes, and cooperatives between these surfaces, thus new surface features in addition to the solid angle may be needed.

## Abbreviations

DSBs: double-stranded DNA binding proteins; SSBs: single-stranded DNA binding proteins; ssDNA: single-stranded DNA; dsDNA: double-stranded DNA; OB-fold: OB (oligonucleotide/oligosaccharide binding) -fold; ACC: accuracy; SN: sensitivity; SP: specificity; F1: F-measured; MCC: Matthews Correlation Coefficient. AUC: area under the receiver operating characteristic curve; TP: true positive; FP: false positive; TN: true negative; FN: false negative.

## Competing interests

The authors declare that they have no competing interests.

## Authors' contributions

W.W., J.L. contributed to the software design and testing. W.W. and X.Z. implemented the software. W.W. and J.L. wrote this paper. All authors read and approved the final manuscript.

## Supplementary Material

Additional file 1 This file contains the complete list of PDB codes for DNA-binding proteins set. Click here for file

Additional file 2 This file describes the classified results of the unknown proteins by the mixed set classifier. Click here for file

## Declarations

The publication costs for this article were funded by the National Science Foundation of China (61272274).

This article has been published as part of *BMC Bioinformatics *Volume 15 Supplement 12, 2014: Selected articles from the IEEE International Conference on Bioinformatics and Biomedicine (BIBM 2013): Bioinformatics. The full contents of the supplement are available online at http://www.biomedcentral.com/bmcbioinformatics/supplements/15/S12.
