# Supplementary material for: Identification of single-stranded and double-stranded dna binding proteins based on protein structure
Source: BMC Bioinformatics. 2014 Nov 6;15(Suppl 12):S4. doi: 10.1186/1471-2105-15-S12-S4 (PMC4243121; doi:10.1186/1471-2105-15-S12-S4)
Supplement: Additional file 1 — This file contains the complete list of PDB codes for DNA-binding proteins set. [file 1471-2105-15-S12-S4-S1.docx]

**S. Table 1 -** **Complete list of PDB codes for DNA-binding protein sets**

| **Types** | **PDB ID** |
| --- | --- |
| **DSBs** | 156D, 1A02, 1A04, 1A28, 1A6Y, 1A73, 1A74, 1AA3, 1AF5, 1AHD, 1AIS, 1AKH, 1AM9, 1AOI, 1APL, 1AU7, 1AWC, 1AZP, 1AZQ, 1B24, 1B3T, 1B8I, 1B8Z, 1BBX, 1BDH, 1BDT, 1BF4, 1BHM, 1BL0, 1BM9, 1BNZ, 1BOW, 1BP7, 1C0W, 1C7U, 1C8C, 1CA6, 1CG7, 1CI4, 1CJG, 1CL3, 1CMA, 1CMO, 1CO1, 1CQT, 1CYQ, 1CZ0, 1CZD, 1D0E, 1D2I, 1D3U, 1D5Y, 1D66, 1D8J, 1D8K, 1DC1, 1DDN, 1DH3, 1DI2, 1DIZ, 1DSZ, 1DUX, 1E3K, 1E3M, 1E50, 1EBM, 1ECR, 1EFA, 1EGW, 1EH6, 1EH7, 1EH8, 1EO4, 1EWQ, 1EWR, 1EXI, 1EXJ, 1EYU, 1F08, 1F0O, 1F4K, 1F5E, 1FJL, 1FJX, 1FOS, 1FSJ, 1FW6, 1FX7, 1FZP, 1G9Y, 1G9Z, 1GAT, 1GAU, 1GDT, 1GLU, 1GU4, 1GVJ, 1H0X, 1H0Y, 1H6F, 1H88, 1H89, 1H8A, 1H9D, 1HCR, 1HJB, 1HJC, 1HMY, 1HRY, 1HRZ, 1HWT, 1I3J, 1IG4, 1IG9, 1IGN, 1IHF, 1IJW, 1IMH, 1IO4, 1IPP, 1IRQ, 1IU3, 1J0R, 1J3E, 1J5N, 1JB7, 1JE8, 1JFI, 1JFS, 1JFT, 1JH9, 1JHB, 1JHZ, 1JIH, 1JJ6, 1JJ8, 1JK1, 1JK2, 1JKO, 1JKP, 1JKQ, 1JKR, 1JR3, 1JWL, 1JX4, 1JXL, 1JY1, 1JYE, 1JYF, 1K78, 1K79, 1K7A, 1KB2, 1KB4, 1KB6, 1KC6, 1KSX, 1KSY, 1L1M, 1LAT, 1LB2, 1LBG, 1LBH, 1LBI, 1LCC, 1LCD, 1LFU, 1LQC, 1M08, 1M5X, 1MD0, 1MDM, 1MHT, 1MJ2, 1MJM, 1MJO, 1MJP, 1MJQ, 1MOW, 1MU7, 1MU9, 1MUH, 1MUS, 1N1J, 1N3E, 1N3F, 1N48, 1N56, 1N6J, 1NA6, 1NFK, 1NG9, 1NJW, 1NJX, 1NJY, 1NJZ, 1NK0, 1NK2, 1NK3, 1NK4, 1NK5, 1NK6, 1NK7, 1NK8, 1NK9, 1NKB, 1NKC, 1NKE, 1NNE, 1NOP, 1NVP, 1NWQ, 1NZB, 1O3Q, 1O3R, 1O3S, 1O3T, 1O4X, 1OCT, 1OFC, 1OH5, 1OH6, 1OH7, 1OH8, 1OMH, 1OOA, 1OSB, 1OSL, 1OTC, 1OUO, 1OUP, 1OWR, 1OYI, 1P51, 1P71, 1P78, 1P7H, 1PDN, 1PER, 1PNR, 1PP7, 1PP8, 1PT3, 1PUE, 1PVI, 1Q0S, 1Q0T, 1Q3U, 1Q5V, 1Q5Y, 1Q9Y, 1QAE, 1QAI, 1QCK, 1QN3, 1QN4, 1QN5, 1QN6, 1QN7, 1QN8, 1QN9, 1QNA, 1QNB, 1QNC, 1QNE, 1QP0, 1QP7, 1QP9, 1QPZ, 1QQA, 1QSS, 1QTM, 1QX0, 1R0N, 1R0O, 1R4I, 1R8E, 1RAM, 1RH6, 1RNL, 1RR8, 1RUO, 1RVC, 1RWZ, 1RXQ, 1RXV, 1RXW, 1RXZ, 1RYR, 1RYS, 1S0M, 1S0N, 1S0O, 1S10, 1S97, 1S9F, 1SD6, 1SD7, 1SFU, 1SI2, 1SI3, 1SKM, 1SMN, 1SQN, 1SR7, 1SRS, 1SX5, 1SX8, 1SXJ, 1T2K, 1T38, 1T39, 1T7P, 1T8E, 1T94, 1T9I, 1T9J, 1TBD, 1TC3, 1TF3, 1TGH, 1TK0, 1TLF, 1TQE, 1TRR, 1TTU, 1TX3, 1U0C, 1U0D, 1U3E, 1U78, 1U8R, 1VFC, 1VKX, 1VOL, 1VTL, 1W0T, 1W0U, 1W7A, 1WB9, 1WBB, 1WBD, 1WTO, 1WTP, 1WTQ, 1WTR, 1WTV, 1WTW, 1WTX, 1X9M, 1X9S, 1X9W, 1XBR, 1XCB, 1XMK, 1XSD, 1XXH, 1YF3, 1YFJ, 1YFL, 1YRN, 1YTF, 1YZ9, 1Z0R, 1ZG1, 1ZG5, 1ZQ3, 1ZQB, 1ZQC, 1ZQD, 1ZQE, 1ZQF, 1ZQG, 1ZQH, 1ZQI, 1ZQJ, 1ZQK, 1ZQL, 1ZQM, 1ZQN, 1ZQO, 1ZQP, 1ZQQ, 1ZQR, 1ZQS, 1ZQT, 1ZTG, 1ZTW, 1ZUC, 2A07, 2A66, 2A6M, 2A6O, 2AC0, 2ACJ, 2ADY, 2AGO, 2AGP, 2AGQ, 2AHI, 2AS5, 2ASD, 2ASJ, 2ASL, 2ATA, 2ATL, 2AU0, 2AXY, 2B5L, 2B5M, 2B5N, 2BCQ, 2BCR, 2BCS, 2BCU, 2BCV, 2BJC, 2BNW, 2BNZ, 2BOP, 2BOW, 2BPF, 2BPG, 2BQ3, 2BQR, 2BQU, 2BR0, 2BSQ, 2BZF, 2C22, 2C28, 2C2D, 2C2E, 2C2R, 2C5R, 2C7A, 2C7O, 2C7P, 2C7Q, 2C7R, 2CAX, 2CDM, 2CGP, 2CKX, 2D45, 2DNJ, 2DPD, 2DPU, 2DRP, 2DY4, 2E42, 2E43, 2ER8, 2ERE, 2ERG, 2EWJ, 2EZ6, 2EZD, 2EZE, 2EZX, 2EZY, 2EZZ, 2F8X, 2FD8, 2FF0, 2FIO, 2FJV, 2FJW, 2FJX, 2FLD, 2FUF, 2GAT, 2GE5, 2GIE, 2GIG, 2GIH, 2GII, 2GIJ, 2GLO, 2GXB, 2H1C, 2H1O, 2H7F, 2H7G, 2H8R, 2HAN, 2HDC, 2HDD, 2HHQ, 2HHS, 2HHT, 2HHU, 2HHV, 2HHW, 2HHX, 2HIN, 2HOS, 2HOT, 2HYE, 2HZA, 2HZV, 2I05, 2I06, 2I13, 2I9G, 2IAJ, 2IPR, 2IRU, 2IRX, 2IRY, 2ISY, 2ISZ, 2IT0, 2ITJ, 2ITL, 2IUS, 2J00, 2J02, 2J6S, 2J6T, 2J6U, 2JEF, 2JEG, 2JEI, 2JEJ, 2JXG, 2JXH, 2JXI, 2K1N, 2KDZ, 2KKF, 2KMK, 2KY8, 2LBS, 2LD5, 2LEX, 2LKX, 2NL8, 2NOG, 2NTC, 2NUF, 2NUG, 2O61, 2O6G, 2O93, 2OG0, 2OH2, 2OST, 2P5O, 2P6R, 2PUF, 2Q2K, 2QHB, 2QNC, 2QNF, 2QSF, 2QSG, 2QSH, 2R1J, 2R2R, 2R2S, 2R2T, 2R2U, 2R5Y, 2R5Z, 2R8J, 2R8K, 2R9L, 2RAM, 2RS6, 2RS7, 2STT, 2STW, 2UU9, 2UUA, 2UUC, 2UVR, 2UVU, 2UVV, 2UVW, 2UXB, 2UXD, 2UZK, 2V4Q, 2V4R, 2V9W, 2VA2, 2VA3, 2VBJ, 2VBL, 2VBN, 2VBO, 2VHG, 2VIC, 2VIH, 2VJU, 2VJV, 2VS7, 2VS8, 2VY1, 2VY2, 2W7O, 2W7P, 2W8K, 2W8L, 2W9A, 2W9B, 2W9C, 2WB2, 2WCC, 2WCW, 2WCZ, 2WDG, 2WDL, 2WIW, 2WIZ, 2WJ0, 2WTU, 2X6N, 2X6S, 2X6U, 2X6V, 2X74, 2X78, 2X9S, 2XB0, 2XE0, 2XM3, 2XQC, 2XRN, 2XRO, 2XSD, 2Y9Y, 2YVE, 2YVH, 2Z3X, 2ZFZ, 2ZOY, 2ZOZ, 3A01, 3A02, 3A03, 3AAF, 3AU2, 3AU6, 3AV1, 3BAM, 3BDN, 3BKZ, 3BRD, 3BRF, 3BRG, 3BUE, 3C2I, 3CAG, 3CBB, 3CIY, 3CLC, 3COQ, 3CVS, 3CVT, 3CW7, 3CWA, 3CWS, 3CWT, 3CWU, 3D0S, 3D6Y, 3D6Z, 3D70, 3D71, 3DFV, 3DFX, 3DLB, 3DLH, 3DLR, 3DO7, 3DZU, 3DZY, 3E00, 3E3Y, 3E40, 3E41, 3E42, 3E43, 3E44, 3E45, 3E54, 3E5Q, 3E5U, 3E6B, 3E6C, 3E6D, 3ECP, 3EEO, 3EIK, 3ERE, 3EXJ, 3EXL, 3EYI, 3F21, 3F22, 3F23, 3FC3, 3FDQ, 3FSI, 3FYA, 3FYL, 3G0Q, 3G2D, 3G4T, 3G5G, 3G6P, 3G6Q, 3G6R, 3G6U, 3G73, 3G8U, 3G8X, 3G97, 3G99, 3G9I, 3G9J, 3G9M, 3G9O, 3G9P, 3GAT, 3GLF, 3GOX, 3GUT, 3GVA, 3GX4, 3GXQ, 3GYH, 3GZ5, 3GZ6, 3GZ8, 3H3U, 3H8O, 3H8R, 3H8X, 3HDD, 3HP6, 3HPO, 3HQF, 3HQG, 3HT3, 3I54, 3I59, 3IAG, 3IH7, 3IKT, 3IKV, 3IL2, 3IMB, 3IN5, 3IRQ, 3IRR, 3IV5, 3IYD, 3JPN, 3JPO, 3JPP, 3JPQ, 3JPR, 3JPS, 3JPT, 3JR9, 3JRA, 3JRB, 3JRC, 3JRD, 3JRE, 3JRF, 3JRG, 3JRH, 3JRI, 3JSO, 3JSP, 3JXB, 3JXC, 3JXD, 3K0S, 3K3R, 3K4G, 3KDE, 3KMD, 3KTQ, 3KTU, 3KXT, 3KYL, 3L1P, 3L2C, 3L4J, 3L4K, 3L8B, 3LNQ, 3LSJ, 3LSP, 3LSR, 3LWH, 3LWI, 3M9Q, 3MDA, 3MDC, 3MFH, 3MFI, 3MFK, 3MGH, 3MGI, 3MKW, 3MKY, 3MKZ, 3MX9, 3MXA, 3MXB, 3MZH, 3N4M, 3N6S, 3N7Q, 3N97, 3NDH, 3OA6, 3OD8, 3ODA, 3ODC, 3ODE, 3OHA, 3OHB, 3OQG, 3OQM, 3OQN, 3OQO, 3ORC, 3OS2, 3OSF, 3OSG, 3OY9, 3OYA, 3P57, 3PKY, 3PML, 3PMN, 3PNC, 3POV, 3PVP, 3PVV, 3PYI, 3PZP, 3Q01, 3Q05, 3Q06, 3Q1M, 3Q2Y, 3Q3D, 3Q5P, 3Q5R, 3Q5S, 3QFQ, 3QMB, 3QMC, 3QMD, 3QMG, 3QMH, 3QMI, 3QOQ, 3QQY, 3QRF, 3QWS, 3QYM, 3QYN, 3RAQ, 3RAX, 3RB0, 3RB3, 3RB4, 3RB6, 3RBD, 3RI4, 3RKQ, 3S8Q, 3SJM, 3SP4, 3SPD, 3SPL, 3SQI, 3SSC, 3SSD, 3SSE, 3SWM, 3SWP, 3TAN, 3TAP, 3TAQ, 3TAR, 3TED, 3THV, 3THW, 3THX, 3THY, 3THZ, 3TI0, 3TQ1, 3U5Z, 3U60, 3U61, 3UFD, 3UKG, 3V68, 3V72, 3VKE, 3VOK, 3ZQC, 3ZVK, 4A08, 4A09, 4A0A, 4A0B, 4A0K, 4A0L, 4A11, 4AAB, 4AAD, 4AAE, 4AAF, 4AAG, 4AVP, 4B06, 4BAC, 4BDY, 4BDZ, 4BE0, 4BE1, 4BE2, 4DM0, 4DQP, 4DQQ, 4DQR, 4DQS, 4DS4, 4DS5, 4DSE, 4DSF, 4DUL, 4E3H, 4E54, 4E7K, 4ENJ, 4ENK, 4ENM, 4ENN, 4ER8, 4ESJ, 4EUW, 4F6M, 4F6N, 4FCY, 4FM9, 4FX0, 4FX4, 4G91, 4G92, 4GAT, 4GHL, 4GZN, 5GAT, 6CRO, 6GAT, 6PAX, 7GAT, 7ICE, 7ICF, 7ICG, 7ICH, 7ICI, 7ICJ, 7ICK, 7ICL, 7ICM, 7ICN, 7ICO, 7ICP, 7ICQ, 7ICR, 7ICS, 7ICT, 7ICU, 7ICV, 8ICA, 8ICB, 8ICC, 8ICE, 8ICF, 8ICG, 8ICH, 8ICI, 8ICJ, 8ICK, 8ICL, 8ICM, 8ICN, 8ICO, 8ICP, 8ICQ, 8ICR, 8ICS, 8ICT, 8ICU, 8ICV, 8ICW, 8ICX, 8ICY, 8ICZ, 9ANT, 9ICA, 9ICB, 9ICC, 9ICE, 9ICF, 9ICG, 9ICH, 9ICI, 9ICJ, 9ICK, 9ICL, 9ICM, 9ICN, 9ICO, 9ICP, 9ICQ, 9ICR, 9ICS, 9ICT, 9ICU, 9ICV, 9ICW, 9ICX, 9ICY, 9MHT. |
| **SSBs** | 1A1V, 1A6B, 1ADU, 1ADV, 1AE2, 1AE3, 1ANV, 1BJ6, 1DMU, 1EQQ, 1EYG, 1F4S, 1FGU, 1GKH, 1GPC, 1GVP, 1H95, 1HAO, 1HAP, 1I7D, 1J4W, 1J5K, 1JE5, 1JMC, 1KAW, 1KDH, 1KIX, 1KXL, 1M06, 1MW8, 1NOY, 1O7I, 1PFS, 1QUQ, 1QVC, 1QZG, 1QZH, 1RB8, 1S3O, 1S40, 1SE8, 1SRU, 1UE1, 1UE5, 1UE6, 1UE7, 1URJ, 1V14, 1V15, 1VQB, 1WOC, 1WTB, 1X0F, 1X3E, 1X3F, 1X3G, 1X5O, 1XF2, 1YUA, 1Z9F, 1ZZK, 2A0I, 2A1K, 2ATQ, 2AYB, 2AYG, 2B29, 2B3G, 2BAN, 2BPA, 2C62, 2CCZ, 2CWA, 2DUD, 2ES2, 2F52, 2FXQ, 2GVA, 2H27, 2HAX, 2HQL, 2I0Q, 2IHE, 2IHF, 2IVH, 2KZM, 2KZZ, 2L45, 2L46, 2LRR, 2PQU, 2PY5, 2PYL, 2UP1, 2VTB, 2VW9, 2VYE, 2W29, 2WAZ, 2WB0, 2WKC, 2WKD, 2WPZ, 2X9U, 3A5U, 3AFP, 3AFQ, 3C94, 3DM3, 3EIV, 3G2C, 3G3Y, 3GP8, 3GPL, 3I1G, 3KJO, 3KJP, 3LGJ, 3MEF, 3N1H, 3N1I, 3N1J, 3N1K, 3N1L, 3NWS, 3PGZ, 3Q8D, 3QK2, 3R9Y, 3R9Z, 3RA0, 3SXU, 3TEK, 3TQY, 3U4V, 3U4Z, 3U50, 3U58, 3U7F, 3U7G, 3U7H, 3UBY, 3UDG, 3UF7, 3UFM, 3ULL, 3ULP, 3VDY, 4A75, 4A8Q, 4A8Y, 4AGH, 4APV, 4DIH, 4DII, 4EXW, 4GS3, 4H5Q. |
| **Unknows protein** | 1A0A, 1A1H, 1A1I, 1A1J, 1A1K, 1A1L, 1A5J, 1A6I, 1A7G, 1AAY, 1ADR, 1AN2, 1AN4, 1AOY, 1ARD, 1ARE, 1ARF, 1AW6, 1B22, 1B28, 1B4O, 1B63, 1B67, 1B69, 1B6W, 1B9M, 1B9N, 1BAZ, 1BBO, 1BDI, 1BGF, 1BGW, 1BHI, 1BHL, 1BI4, 1BIA, 1BJ0, 1BJZ, 1BKN, 1BL3, 1BM8, 1BVS, 1BW5, 1BW6, 1BY4, 1BY9, 1C1K, 1C20, 1C6V, 1C8G, 1C9B, 1CDW, 1CDZ, 1CG0, 1CI6, 1CLD, 1CLQ, 1CS9, 1CT6, 1CVQ, 1CW8, 1CWZ, 1CY8, 1D1M, 1D3Y, 1D4U, 1D5V, 1D8B, 1D9N, 1D9X, 1D9Z, 1DBD, 1DHM, 1DML, 1DP3, 1DP7, 1DPS, 1DPU, 1DU0, 1DU6, 1DV0, 1E17, 1E3F, 1E4H, 1E7D, 1E9N, 1EA4, 1EA6, 1EAN, 1EAO, 1EAQ, 1EE8, 1EG2, 1EIJ, 1ENH, 1EO3, 1ETO, 1ETQ, 1ETV, 1ETW, 1ETX, 1ETY, 1EX4, 1EXE, 1EYF, 1F1E, 1F1Z, 1F2I, 1F2R, 1F30, 1F33, 1F4I, 1F4R, 1F5T, 1F6O, 1F6V, 1F9F, 1F9N, 1FBQ, 1FBS, 1FBU, 1FIA, 1FIP, 1FIU, 1FLI, 1FTT, 1FTZ, 1FU1, 1FWZ, 1FYK, 1FYL, 1FYM, 1G2D, 1G2F, 1G2H, 1G3S, 1G3T, 1G3W, 1G50, 1G5H, 1G5I, 1G6N, 1G8E, 1GCB, 1GCC, 1GE8, 1GHT, 1GTW, 1GU5, 1GUU, 1GV2, 1GV5, 1GVD, 1GXC, 1GXP, 1GXQ, 1GZH, 1H0M, 1H1J, 1H2I, 1H3L, 1H5P, 1H7U, 1H9G, 1H9T, 1HCP, 1HCQ, 1HD7, 1HDP, 1HKQ, 1HKS, 1HKT, 1HLO, 1HLV, 1HNR, 1HNS, 1HOM, 1HSJ, 1HU8, 1HUO, 1HUU, 1HUZ, 1HW1, 1HX7, 1HXD, 1HYV, 1I11, 1I5Z, 1I6X, 1IDY, 1IDZ, 1IF1, 1IFY, 1IG1, 1IG6, 1IHV, 1IHW, 1IN4, 1IN5, 1IN6, 1IN7, 1IN8, 1IR6, 1IRZ, 1ISQ, 1ITY, 1IUF, 1IV6, 1IXC, 1IXY, 1IYJ, 1IYM, 1IZ1, 1IZ4, 1IZ5, 1J07, 1J1J, 1J1V, 1J2F, 1J3C, 1J3D, 1J3X, 1J4P, 1J53, 1J59, 1J7K, 1J9I, 1J9N, 1JEQ, 1JEY, 1JGS, 1JIC, 1JJH, 1JJR, 1JRE, 1JSP, 1JT0, 1JTS, 1JTX, 1JUN, 1JUP, 1JUS, 1JXS, 1K0P, 1K18, 1K1V, 1K6I, 1K6J, 1K6X, 1K82, 1K8G, 1K99, 1KAF, 1KCA, 1KEG, 1KFT, 1KGS, 1KKX, 1KN0, 1KN5, 1KNU, 1KQ8, 1KU5, 1KU9, 1KW4, 1KZ0, 1KZ2, 1KZ5, 1KZY, 1L1O, 1L3A, 1L3L, 1L3R, 1L8H, 1L8I, 1L8Q, 1L8Y, 1L8Z, 1LE8, 1LJ9, 1LJM, 1LMB, 1LNW, 1LPV, 1LR1, 1LRP, 1LWM, 1LWS, 1LWW, 1LX8, 1M36, 1M3H, 1M55, 1M5R, 1M6I, 1M6U, 1MB1, 1MBE, 1MBF, 1MBG, 1MBH, 1MBJ, 1MBK, 1MDY, 1MH3, 1MH4, 1MI7, 1MIJ, 1MIU, 1MM2, 1MM3, 1MM8, 1MN4, 1MNM, 1MNT, 1MOJ, 1MP9, 1MSE, 1MSF, 1MSZ, 1MUL, 1MY5, 1N00, 1N03, 1N0W, 1N5G, 1N83, 1NEQ, 1NER, 1NFH, 1NFJ, 1NGM, 1NGN, 1NH2, 1NH9, 1NI8, 1NLI, 1NR3, 1NTC, 1NYS, 1NZP, 1O57, 1O7L, 1ODD, 1OIS, 1OJL, 1OLO, 1OMQ, 1ON1, 1ON2, 1OQJ, 1OR7, 1OSV, 1OV9, 1OY3, 1P1A, 1P4D, 1P4E, 1P4W, 1P6R, 1P7A, 1P7D, 1P7I, 1P7J, 1P92, 1P94, 1PA6, 1PCZ, 1PFE, 1PFR, 1PGZ, 1PH1, 1PH2, 1PH3, 1PH4, 1PH5, 1PH6, 1PH7, 1PH8, 1PH9, 1PHJ, 1PIQ, 1PL5, 1POG, 1POU, 1PRU, 1PRV, 1PVE, 1PYC, 1PZU, 1Q1V, 1Q87, 1Q88, 1Q89, 1Q8H, 1Q9X, 1QAJ, 1QEY, 1QGP, 1QK9, 1QP4, 1QPI, 1QQB, 1QRV, 1QRY, 1QSY, 1QVP, 1QVT, 1QVU, 1QWT, 1QZQ, 1R05, 1R1U, 1R1V, 1R22, 1R23, 1R36, 1R4O, 1R4R, 1R5K, 1R63, 1R71, 1R7J, 1R7M, 1R8D, 1R8H, 1R8P, 1RB4, 1RB5, 1RB6, 1REA, 1RES, 1RET, 1RFF, 1RFI, 1RG1, 1RG2, 1RGD, 1RGT, 1RGU, 1RH0, 1RI7, 1RIF, 1RIY, 1RMD, 1RUN, 1RW2, 1RYU, 1RZN, 1RZR, 1RZS, 1S3N, 1S6M, 1S7E, 1S7O, 1S7Z, 1S9K, 1SA3, 1SAN, 1SAP, 1SAX, 1SD4, 1SEU, 1SFE, 1SKX, 1SM4, 1SP1, 1SP2, 1SQ8, 1SSO, 1STZ, 1SUT, 1SUZ, 1SV0, 1SV4, 1SVC, 1SZP, 1T0F, 1T23, 1T2R, 1T2S, 1T33, 1T4W, 1T7A, 1TA8, 1TBP, 1TBX, 1TF6, 1TN9, 1TNS, 1TNT, 1TP4, 1TRO, 1TXY, 1TZW, 1TZY, 1U2W, 1U36, 1U3J, 1U3Y, 1U3Z, 1U41, 1U42, 1U85, 1U86, 1U94, 1U98, 1U99, 1U9N, 1U9O, 1UB4, 1UBD, 1UD9, 1UDV, 1UFI, 1UHL, 1UKL, 1UL1, 1UL4, 1UL5, 1ULY, 1UMQ, 1UOL, 1USS, 1UST, 1UT7, 1UT8, 1UTX, 1UVH, 1UW0, 1UXC, 1UXD, 1V06, 1V1Q, 1V63, 1V64, 1VA1, 1VA2, 1VA3, 1VD7, 1VD8, 1VD9, 1VDA, 1VDB, 1VEI, 1VEL, 1VEQ, 1VF9, 1VHI, 1VJF, 1VND, 1VOK, 1VQC, 1VQD, 1VQE, 1VQG, 1VQH, 1VQI, 1VQJ, 1VTN, 1VTO, 1VYJ, 1VYM, 1W3S, 1W5S, 1W5T, 1W60, 1WCV, 1WE9, 1WEE, 1WEM, 1WEN, 1WEO, 1WEP, 1WES, 1WET, 1WEU, 1WEW, 1WG2, 1WG6, 1WGF, 1WGX, 1WH5, 1WH7, 1WI3, 1WID, 1WIJ, 1WIZ, 1WJ0, 1WJ2, 1WJV, 1WLZ, 1WNE, 1WNK, 1WNM, 1WNN, 1WPK, 1WRP, 1WRQ, 1WTU, 1WVL, 1WWX, 1WXL, 1X2I, 1X3C, 1X41, 1X51, 1X57, 1X58, 1X5W, 1X65, 1X6E, 1X6F, 1X6H, 1X9N, 1XBF, 1XC8, 1XCV, 1XD7, 1XFP, 1XG1, 1XGK, 1XMS, 1XMV, 1XNA, 1XNT, 1XO5, 1XP8, 1XPA, 1XPX, 1XSL, 1XSN, 1XSP, 1XSX, 1XTK, 1XV9, 1XVP, 1XWR, 1XX8, 1XXA, 1XXB, 1XXC, 1XYI, 1Y0J, 1Y0U, 1Y1V, 1Y23, 1Y5R, 1Y6U, 1Y9X, 1YCQ, 1YCZ, 1YD0, 1YD1, 1YD2, 1YD3, 1YD4, 1YD5, 1YD6, 1YDX, 1YHA, 1YHB, 1YIO, 1YJM, 1YLF, 1YNW, 1YNX, 1YQA, 1YQM, 1YS6, 1YS7, 1YSE, 1YTB, 1YUI, 1YUJ, 1Z19, 1Z1B, 1Z1G, 1Z3E, 1Z4H, 1Z6R, 1Z91, 1Z9C, 1ZAE, 1ZAY, 1ZEL, 1ZGW, 1ZI0, 1ZM5, 1ZME, 1ZNF, 1ZNM, 1ZNS, 1ZP7, 1ZR9, 1ZRC, 1ZRD, 1ZRE, 1ZRJ, 1ZS3, 1ZT9, 1ZTT, 1ZUJ, 1ZVV, 1ZZF, 2A11, 2A1I, 2A1J, 2A3S, 2AA2, 2AA5, 2AA6, 2AA7, 2ADL, 2ADN, 2ADR, 2AIF, 2AJE, 2ALC, 2AN7, 2AQL, 2AUH, 2AUW, 2AW6, 2AWI, 2AXL, 2AY0, 2AYD, 2AYE, 2AYR, 2B0D, 2B0E, 2B0L, 2B21, 2B2N, 2B2U, 2B8A, 2B8K, 2BA3, 2BAM, 2BDE, 2BEJ, 2BEK, 2BEO, 2BGC, 2BH8, 2BIM, 2BIN, 2BIO, 2BIP, 2BIQ, 2BJ1, 2BJ3, 2BJ8, 2BJ9, 2BJY, 2BK6, 2BKC, 2BKE, 2BKY, 2BNK, 2BYK, 2BYM, 2BZE, 2C2F, 2C2J, 2C2U, 2C6R, 2C96, 2C98, 2C99, 2C9C, 2CA9, 2CAD, 2CBM, 2CG4, 2CGN, 2CH0, 2CHG, 2CHP, 2CHQ, 2CHV, 2CJJ, 2CMP, 2COB, 2CPG, 2CPJ, 2CQ2, 2CQX, 2CRG, 2CRJ, 2CRU, 2CS1, 2CSF, 2CU7, 2CUF, 2CVF, 2CVH, 2CVR, 2CXY, 2CYY, 2CZR, 2D1H, 2D2W, 2D5R, 2D7E, 2D7G, 2D7H, 2D7L, 2D8M, 2D9A, 2D9H, 2DA1, 2DA2, 2DA3, 2DA4, 2DA5, 2DA6, 2DA7, 2DAO, 2DB7, 2DBF, 2DDG, 2DFL, 2DGC, 2DGZ, 2DH5, 2DIG, 2DIM, 2DIN, 2DL6, 2DLK, 2DMD, 2DMP, 2DMQ, 2DMS, 2DMT, 2DMU, 2DQB, 2DQR, 2DS0, 2DT5, 2E1N, 2E1O, 2E5R, 2E6R, 2E7N, 2E7O, 2E7W, 2E7X, 2EBI, 2EBL, 2EBW, 2EBY, 2ECC, 2ECS, 2EF8, 2EFN, 2EFO, 2EFP, 2EFQ, 2EH9, 2EL4, 2EL5, 2EL6, 2ELH, 2ELY, 2ELZ, 2EM0, 2EO0, 2EPB, 2EQJ, 2EQY, 2EVF, 2EVG, 2EVH, 2EVI, 2EVJ, 2EW0, 2EWT, 2EZF, 2EZG, 2EZH, 2EZI, 2EZK, 2EZL, 2F2E, 2F5C, 2F5D, 2F5E, 2F5F, 2F7N, 2F7T, 2FBK, 2FC7, 2FD5, 2FDC, 2FE3, 2FIP, 2FKI, 2FMQ, 2FMY, 2FNP, 2FPH, 2FPL, 2FQ4, 2FU4, 2FWO, 2FWR, 2FZ4, 2FZL, 2G3R, 2G7O, 2G99, 2G9A, 2G9E, 2G9W, 2GCC, 2GEQ, 2GFU, 2GMG, 2GN5, 2GPE, 2GUI, 2GX5, 2GZU, 2GZW, 2H1L, 2H40, 2H5X, 2H6B, 2H6C, 2H9U, 2HAP, 2HGV, 2HI2, 2HI5, 2HIL, 2HIO, 2HKV, 2HKX, 2HT0, 2HTS, 2HUE, 2HW0, 2HX0, 2HYJ, 2HZD, 2HZM, 2HZT, 2I1Q, 2I4J, 2I4Z, 2I5L, 2I5M, 2I5O, 2I5W, 2ICP, 2ICT, 2IEF, 2IIC, 2IJG, 2IJL, 2IJX, 2IKS, 2ING, 2IO4, 2ITD, 2IUU, 2IWJ, 2IYN, 2J0V, 2J10, 2J11, 2J2S, 2J4D, 2J7Y, 2J85, 2J9P, 2JA6, 2JB9, 2JBA, 2JCG, 2JD3, 2JES, 2JEX, 2JF9, 2JJ7, 2JK3, 2JMP, 2JMW, 2JNW, 2JOX, 2JP9, 2JPA, 2JPB, 2JPC, 2JPD, 2JR1, 2JSP, 2JTM, 2JUH, 2JUL, 2JUO, 2JV0, 2JV3, 2JX3, 2JYI, 2JYW, 2K0C, 2K27, 2K3Y, 2K40, 2K4B, 2K5V, 2K6G, 2K6L, 2K75, 2K7S, 2K86, 2K8F, 2K9I, 2K9N, 2K9S, 2KA6, 2KBY, 2KDK, 2KEB, 2KEI, 2KEJ, 2KEK, 2KFS, 2KFT, 2KHL, 2KHQ, 2KI2, 2KIU, 2KIW, 2KJ8, 2KJ9, 2KJC, 2KK0, 2KKO, 2KKP, 2KKV, 2KM4, 2KN8, 2KNG, 2KNH, 2KQD, 2KRF, 2KW3, 2KWQ, 2KZ5, 2L0K, 2L1G, 2L1P, 2L3N, 2L3R, 2L49, 2L4A, 2L4M, 2L66, 2L6Y, 2L6Z, 2L75, 2L7M, 2L7Z, 2L8D, 2L8E, 2L8N, 2L92, 2L93, 2L9R, 2LAU, 2LCV, 2LDU, 2LE4, 2LF7, 2LF8, 2LFH, 2LGG, 2LGK, 2LI6, 2LJ6, 2LK2, 2LKP, 2LLH, 2LM1, 2LQ6, 2LSG, 2LSS, 2LSY, 2LTT, 2LUA, 2LW1, 2LY4, 2LY9, 2NMU, 2NOF, 2NOL, 2NOV, 2NP2, 2NTI, 2NX4, 2O03, 2O3C, 2O49, 2O4A, 2O7H, 2O7M, 2O7T, 2O8B, 2O8C, 2O8D, 2O8E, 2O8F, 2O97, 2O99, 2O9A, 2O9I, 2O9L, 2OBP, 2OCJ, 2OD5, 2OKF, 2OVG, 2OVH, 2OVM, 2OWO, 2OWY, 2OXL, 2OXO, 2OXV, 2OZ6, 2OZ9, 2OZE, 2P2R, 2P2U, 2P4W, 2P52, 2P5K, 2P5L, 2P5M, 2P6U, 2P81, 2PBX, 2PCX, 2PEX, 2PG4, 2PI0, 2PI2, 2PKH, 2PMH, 2PN6, 2PNH, 2PQA, 2PUA, 2PUB, 2PUG, 2PY9, 2Q2T, 2Q79, 2QDG, 2QDH, 2QE4, 2QFJ, 2QIB, 2QL2, 2QLC, 2QMV, 2QNQ, 2QPW, 2QPY, 2QR9, 2QRX, 2QSE, 2QSJ, 2QSX, 2QTU, 2QU7, 2QUQ, 2QV0, 2QVA, 2QWT, 2QWW, 2QXM, 2QXS, 2QZ8, 2QZO, 2R2W, 2R56, 2R58, 2R5A, 2R5M, 2R6W, 2R6Y, 2R7Z, 2RAE, 2RAS, 2RBA, 2RBF, 2RDP, 2REB, 2REK, 2RGR, 2RGV, 2RGY, 2RH3, 2RHI, 2RHX, 2RHY, 2RHZ, 2RI2, 2RI3, 2RI5, 2RJC, 2RJE, 2RNJ, 2RNN, 2RO4, 2RO5, 2ROH, 2RPC, 2RQP, 2RRD, 2TBD, 2TDX, 2TRT, 2UVP, 2UXC, 2UXI, 2UXP, 2UYF, 2V0R, 2V0X, 2V1C, 2V2T, 2V79, 2V85, 2V89, 2VBW, 2VBX, 2VBY, 2VBZ, 2VC0, 2VC1, 2VEQ, 2VF7, 2VF8, 2VI6, 2VKV, 2VL6, 2VPK, 2VPR, 2VQC, 2VRZ, 2VSF, 2VUK, 2VUM, 2VUU, 2VXX, 2W1R, 2W1T, 2W24, 2W25, 2W74, 2W7N, 2WBR, 2WBS, 2WBT, 2WBU, 2WC2, 2WG5, 2WG6, 2WGX, 2WIU, 2WP0, 2WPU, 2WPY, 2WQ1, 2WQ2, 2WQ3, 2WQI, 2WQJ, 2WTT, 2WYQ, 2X35, 2X6O, 2X9D, 2XBM, 2XCP, 2XD7, 2XDI, 2XIU, 2XIW, 2XJ3, 2XJ9, 2XMA, 2XNH, 2XNK, 2XO6, 2XRL, 2XRZ, 2XWC, 2Y3M, 2Y75, 2Y7P, 2Y8Q, 2YGV, 2YQE, 2YRM, 2YRQ, 2YT5, 2YTV, 2YTX, 2YTY, 2YUM, 2YVA, 2YW6, 2YW7, 2YX4, 2YX7, 2Z33, 2Z43, 2Z47, 2Z4P, 2Z4Q, 2Z4R, 2Z4S, 2Z6A, 2Z6K, 2Z7C, 2Z8H, 2Z8U, 2Z90, 2ZAS, 2ZBE, 2ZBK, 2ZCM, 2ZCN, 2ZCW, 2ZDS, 2ZFX, 2ZHG, 2ZHH, 2ZK0, 2ZK2, 2ZK3, 2ZK4, 2ZK6, 2ZKZ, 2ZL9, 2ZMH, 2ZMI, 2ZMJ, 2ZNO, 2ZNQ, 2ZNS, 2ZNY, 2ZNZ, 2ZQE, 2ZTC, 2ZTD, 2ZTE, 2ZUB, 2ZUC, 2ZVT, 2ZVV, 2ZVW, 2ZWJ, 2ZXJ, 3A1J, 3A2I, 3A5T, 3A77, 3A78, 3ADS, 3ADU, 3ADV, 3ADW, 3ADX, 3AFR, 3AK8, 3AK9, 3AL2, 3AL3, 3ALC, 3AN2, 3ANG, 3ANP, 3AO1, 3AO2, 3AO3, 3AO4, 3AO5, 3AOH, 3AQK, 3AQM, 3AQQ, 3AQS, 3AQT, 3ASK, 3ASL, 3ASM, 3AUO, 3AV2, 3AXJ, 3AXY, 3B02, 3B0B, 3B0C, 3B0D, 3B20, 3B2N, 3B39, 3B3K, 3B5R, 3B65, 3B66, 3B67, 3B68, 3B6A, 3B6C, 3B81, 3B84, 3BAQ, 3BC4, 3BCG, 3BDD, 3BDP, 3BEJ, 3BFN, 3BFU, 3BHQ, 3BIM, 3BIT, 3BKT, 3BNI, 3BOS, 3BPV, 3BPX, 3BQO, 3BQY, 3BR1, 3BR2, 3BR3, 3BR5, 3BR6, 3BRO, 3BRQ, 3BRU, 3BS1, 3BS3, 3BSB, 3BTC, 3BTI, 3BTJ, 3BTL, 3BTP, 3BTS, 3BTU, 3BTV, 3BU8, 3BUA, 3BVQ, 3BWG, 3BXE, 3BXF, 3BXG, 3BXH, 3BY6, 3BZJ, 3C07, 3C0Q, 3C1D, 3C1Y, 3C1Z, 3C21, 3C23, 3C25, 3C2G, 3C3W, 3C46, 3C4I, 3C57, 3C58, 3C5F, 3C5G, 3C6A, 3C9C, 3C9K, 3CB5, 3CB6, 3CDL, 3CJD, 3CJN, 3CJW, 3CLD, 3CLK, 3CLN, 3CLO, 3CMM, 3CNB, 3COL, 3CQV, 3CRH, 3CRJ, 3CRP, 3CS8, 3CTB, 3CTP, 3CUO, 3CWR, 3CXU, 3CZ5, 3CZ6, 3D05, 3D06, 3D07, 3D08, 3D09, 3D1N, 3D6W, 3D8A, 3D8U, 3D90, 3DBI, 3DCF, 3DCT, 3DCU, 3DEE, 3DEU, 3DEW, 3DI8, 3DJL, 3DKX, 3DNV, 3DPJ, 3DR1, 3DSH, 3DT3, 3DTK, 3E0C, 3E1S, 3E1U, 3E3M, 3E4U, 3E6M, 3E7Q, 3E97, 3E9F, 3ECH, 3EET, 3EGQ, 3EI1, 3EI2, 3EI3, 3EI4, 3EMQ, 3EN2, 3EPG, 3EQX, 3ER9, 3ERC, 3ET0, 3ET1, 3ET2, 3ET3, 3ETL, 3EU7, 3EUL, 3EUS, 3EW9, 3EWA, 3EY1, 3EYZ, 3EZ2, 3EZ5, 3EZ6, 3EZ7, 3EZ9, 3EZF, 3F1B, 3F1N, 3F1P, 3F1Z, 3F27, 3F2B, 3F2C, 3F2D, 3F3X, 3F5R, 3F6C, 3F6P, 3F6V, 3F6W, 3F72, 3F73, 3F8L, 3F8N, 3FAJ, 3FAU, 3FBR, 3FD3, 3FD9, 3FEI, 3FEJ, 3FGM, 3FHG, 3FHW, 3FHZ, 3FIS, 3FIW, 3FJA, 3FJD, 3FJJ, 3FJK, 3FK7, 3FKC, 3FMS, 3FMY, 3FPN, 3FRQ, 3FSP, 3FSQ, 3FT7, 3FUR, 3FWE, 3FYM, 3FZV, 3G00, 3G0R, 3G1C, 3G1L, 3G1M, 3G1O, 3G38, 3G3C, 3G56, 3G65, 3G85, 3G8I, 3G8O, 3G9E, 3GA6, 3GA8, 3GBG, 3GBV, 3GCC, 3GD2, 3GE4, 3GEZ, 3GF2, 3GFI, 3GFJ, 3GFL, 3GFM, 3GIO, 3GJP, 3GL6, 3GLB, 3GN5, 3GP4, 3GPM, 3GPN, 3GPV, 3GRA, 3GTM, 3GUD, 3GUV, 3GV6, 3GW2, 3GYB, 3GYD, 3H0A, 3H0D, 3H0G, 3H1D, 3H40, 3H4L, 3H5O, 3H5T, 3H7W, 3H82, 3HC5, 3HC6, 3HE0, 3HGG, 3HH0, 3HHF, 3HHG, 3HI2, 3HIF, 3HJF, 3HK2, 3HLV, 3HM1, 3HM5, 3HM9, 3HMF, 3HNM, 3HO1, 3HOS, 3HOT, 3HQR, 3HQU, 3HRS, 3HRT, 3HRU, 3HS3, 3HSE, 3HSF, 3HSR, 3HTA, 3HTH, 3HTI, 3HTJ, 3HTN, 3HTS, 3HUG, 3HVL, 3HXM, 3I1L, 3I2E, 3I2Z, 3I38, 3I49, 3I4P, 3I7K, 3I7L, 3IA6, 3IAO, 3IAY, 3IGC, 3IGD, 3IGL, 3IGM, 3IH4, 3IHQ, 3IHU, 3II2, 3II3, 3II6, 3IIF, 3IKM, 3ILD, 3ILE, 3IO5, 3IP8, 3IPQ, 3IPU, 3IU5, 3IU6, 3IVP, 3IWZ, 3IX3, 3IX4, 3IX8, 3J0K, 3JSJ, 3JTZ, 3JU0, 3JUA, 3JV4, 3JV5, 3JV6, 3JY6, 3JZB, 3K0L, 3K1M, 3K1N, 3K1P, 3K2A, 3K2N, 3K2Z, 3K44, 3K4H, 3K4T, 3K4X, 3K57, 3K58, 3K59, 3K75, 3K7Z, 3K8A, 3K98, 3KEO, 3KEQ, 3KHK, 3KHT, 3KJV, 3KK1, 3KK2, 3KK3, 3KKC, 3KKD, 3KKE, 3KKR, 3KKS, 3KLW, 3KMP, 3KMR, 3KN1, 3KNT, 3KNW, 3KO2, 3KOJ, 3KOR, 3KOV, 3KP1, 3KYS, 3KZ5, 3KZ7, 3KZ8, 3KZ9, 3L03, 3L0E, 3L15, 3L2Q, 3L2R, 3L2U, 3L2V, 3L2W, 3L3U, 3L3V, 3L41, 3L51, 3L57, 3L5Z, 3LA2, 3LA3, 3LA7, 3LAJ, 3LAP, 3LDA, 3LDK, 3LDS, 3LFP, 3LGD, 3LHK, 3LIS, 3LJL, 3LJW, 3LMP, 3LOC, 3LQC, 3LSG, 3LV3, 3LWF, 3LWJ, 3LX1, 3LX2, 3LY7, 3LYR, 3LZ8, 3M03, 3M0E, 3M1E, 3M36, 3M37, 3M48, 3M4A, 3M52, 3M5B, 3M66, 3M6K, 3M6R, 3M6Z, 3M7G, 3M89, 3M8E, 3M8F, 3M8K, 3M8V, 3M9A, 3M9E, 3M9M, 3M9N, 3M9O, 3MAJ, 3MAQ, 3MCP, 3ME9, 3MEA, 3MET, 3MEU, 3MEV, 3MEW, 3MNL, 3MSK, 3MU6, 3MUJ, 3N4P, 3N4Q, 3N50, 3N89, 3N8B, 3NCT, 3NFH, 3NFI, 3NGQ, 3NHZ, 3NM7, 3NNN, 3NNS, 3NO0, 3NO7, 3NQJ, 3NQU, 3NR7, 3NWT, 3NXC, 3NZL, 3O27, 3O3V, 3O4N, 3O4Q, 3O74, 3O75, 3O8G, 3O9X, 3OC3, 3OD2, 3OIO, 3OIQ, 3OKG, 3OKT, 3OLC, 3OM4, 3OMY, 3ON0, 3OS0, 3OS1, 3OSN, 3OVN, 3OXF, 3OXG, 3OXL, 3OY3, 3P7N, 3P83, 3P87, 3P8B, 3P91, 3P9A, 3PBA, 3PC6, 3PC7, 3PC8, 3PF4, 3PF5, 3PGE, 3PGG, 3PLQ, 3PM1, 3PV8, 3PX0, 3PX4, 3PX6, 3PXP, 3Q0F, 3Q22, 3Q23, 3Q24, 3Q4F, 3Q9S, 3Q9V, 3QAO, 3QBI, 3QBK, 3QBM, 3QBT, 3QLP, 3QO2, 3QO3, 3QOD, 3QOE, 3QOP, 3QP1, 3QP2, 3QP4, 3QP5, 3QP6, 3QP8, 3QPS, 3QQ6, 3QQA, 3QSI, 3QU3, 3QU6, 3QVG, 3R0J, 3R4K, 3R8F, 3R8I, 3RCO, 3RCW, 3RCZ, 3RDI, 3REU, 3RH2, 3RHI, 3RIR, 3RJP, 3RKW, 3RKX, 3RKY, 3RL6, 3RLN, 3RLO, 3RMB, 3RMD, 3RMH, 3RMP, 3RN2, 3RN5, 3RNU, 3ROU, 3RPQ, 3RSN, 3RWR, 3RYP, 3RYR, 3S32, 3S3M, 3S3N, 3S3O, 3S4W, 3S4Z, 3S51, 3S57, 3S5R, 3S5U, 3S7W, 3SC3, 3SDG, 3SFI, 3SHO, 3SI8, 3SIA, 3SIB, 3SJS, 3SON, 3SR2, 3SSW, 3SSX, 3SXK, 3SXM, 3SXY, 3SXZ, 3SZP, 3T72, 3T79, 3T7R, 3TB6, 3THN, 3THO, 3TOC, 3TOE, 3TP0, 3TRB, 3TUO, 3TZD, 3U21, 3U33, 3U44, 3U4Q, 3U5L, 3U6Y, 3U7E, 3UB2, 3UBT, 3UC1, 3UFC, 3UGM, 3UGO, 3UGP, 3ULJ, 3ULQ, 3ULX, 3UN0, 3UQ2, 3UQ8, 3UQZ, 3US0, 3US1, 3US2, 3UWX, 3UX8, 3UXG, 3V20, 3V21, 3V4G, 3V60, 3V61, 3V62, 3V6G, 3V6P, 3V6T, 3V7F, 3V9R, 3VD0, 3VD1, 3VD2, 3VH5, 3VH6, 3VIB, 3VJZ, 3VK0, 3VK7, 3VK8, 3VKX, 3VNC, 3VPR, 3VX6, 3W03, 3WRP, 3ZNF, 3ZQ7, 3ZQJ, 3ZQL, 3ZQM, 3ZQO, 3ZQP, 3ZQQ, 3ZTH, 4A2U, 4A3N, 4A3V, 4A5N, 4AA6, 4ACO, 4AYA, 4B4C, 4BBQ, 4DC9, 4DFC, 4DG7, 4DHX, 4DQ2, 4DQI, 4DQY, 4DRA, 4DRB, 4DT1, 4DUG, 4E07, 4E09, 4E0D, 4E1P, 4E1R, 4E2I, 4E5Z, 4E7H, 4E7I, 4E7J, 4E7L, 4EL5, 4EQ6, 4EV0, 4EW0, 4EXG, 4FBQ, 4FIS, 4FJO, 4FMR, 4G20, 4G21, 4G2H, 4G63, 4G7H, 4G7O, 4G7Z, 4GFH, 4GG4, 4GOR, 4GWQ, 4H79, 4H7A, 4H9N, 4H9O, 4H9P, 4H9Q, 4H9R, 4H9S, 4HD0, 4TMK, 4ZNF, 5CRO, 5KTQ, 5ZNF, 7ZNF. |
